# Physical and Mechanical Properties of Particleboards Made from Furfurylated Rattan Particles

**DOI:** 10.3390/polym18091031

**Published:** 2026-04-24

**Authors:** Mahdi Mubarok, Nela Rahmati Sari, Lukmanul Hakim Zaini, Purwantiningsih Sugita, Muhammad Adly Rahandi Lubis, Imam Busyra Abdillah, Abdus Syukur, Eko Setio Wibowo, Ignasia Maria Sulastiningsih, Jingjing Liao, Dede Hermawan, Philippe Gérardin, Ioanna A. Papadopoulou, Antonios N. Papadopoulos

**Affiliations:** 1 Department of Forest Products, Faculty of Forestry and Environment, IPB University, Bogor 16680, Indonesia; nelarahmatisari@gmail.com (N.R.S.); lukmanhz@apps.ipb.ac.id (L.H.Z.); ibusyra.a@gmail.com (I.B.A.); abdssykr18@gmail.com (A.S.); dedehe@apps.ipb.ac.id (D.H.); 2 Center for Sustainable Forest Development, Bogor 16118, Indonesia; 3 Department of Chemistry, Faculty of Mathematics and Natural Sciensces, IPB University, Bogor 16680, Indonesia; purwantiningsih@apps.ipb.ac.id; 4 Research Center for Biomass and Bioproducts, National Research and Innovation Agency (BRIN), South Tangerang 15314, Indonesia; muha142@brin.go.id (M.A.R.L.); ekos009@brin.go.id (E.S.W.); igna009@brin.go.id (I.M.S.); 5 Key Laboratory of Vegetable Biology of Yunnan Province, College of Landscape and Horticulture, Yunnan Agricultural University, Kunming 650201, China; jingjing_liao@hotmail.com; 6 LERMAB, INRAE, Université de Lorraine, 54000 Nancy, France; philippe.gerardin@univ-lorraine.fr; 7 Department of Chemistry, Aristotle University of Thessaloniki, 54124 Thessaloniki, Greece; papad.ioanna@chem.auth.gr; 8 Department of Natural Environment and Climate Resilience, Democritus University of Thrace, 66100 Drama, Greece

**Keywords:** furfuryl alcohol, mechanical properties, particleboard, physical properties, rattan

## Abstract

The limited availability of high-quality timber and the increasing demand for wood-based panels have encouraged the exploration of alternative and sustainable lignocellulosic resources. Rattan waste is abundant in Indonesia; however, its low mechanical strength and limited durability restrict its direct application in composite materials. This study investigated the effect of furfuryl alcohol (FA) modification and different adhesive systems on the performance of rattan-based particleboard. Rattan particles were immersed in FA for 24 h and used to produce particleboards (300 × 300 × 10 mm) bonded with phenol formaldehyde (PF), melamine formaldehyde (MF), and urea formaldehyde (UF) adhesives at a resin content of 12%. The boards were manufactured under controlled hot pressing conditions and conditioned for 14 days prior to testing. Furfurylation significantly improved dimensional stability by reducing moisture content, water absorption, thickness swelling, and leaching, with anti-swelling efficiency values ranging from 43.25% to 71.06%. Some selected mechanical properties, including internal bonding strength, hardness, and screw holding power, were also enhanced. However, the modification showed limited influence on the modulus of elasticity and, in some cases, reduced the modulus of rupture. Among the adhesive systems, MF-bonded boards exhibited the most balanced mechanical performance. Furfurylation also produced darker and more uniform board surfaces. These findings indicate that furfurylated rattan particleboards are suitable for non-structural and decorative applications.

## 1. Introduction

The further growth of Indonesia’s furniture and construction sector has led to constant growth in the demand for engineered wood panels, especially particleboard. However, the utilization of natural timber is increasingly constrained by limited supplies of high-quality wood and escalating environmental concerns associated with unsustainable forest exploitation. Deforestation and forest degradation have significantly reduced the availability of raw materials, with more than 261,000 ha of forest lost in 2024 [1]. At present, over 75% of the national wood supply originates from plantation forests, which are generally characterized by inferior quality, high susceptibility to biological degradation, and low physical and mechanical performance [2]. These conditions highlight the urgent need for alternative and more sustainable lignocellulosic resources.

The rich supply of rattan waste has a great potential to be used as an alternative raw material in particleboard manufacturing. Indonesia supplies approximately 80% of the global rattan market, with major production centers in Kalimantan, Sumatra, Sulawesi, and Papua, including West Sumatra as a significant contributor [3]. Despite its abundance, a substantial portion of rattan biomass generated by the furniture and handicraft industries remains underutilized. As a fast-growing tropical resource, rattan offers considerable potential as a renewable raw material for wood-based composites. Nevertheless, its intrinsic limitations, particularly its low mechanical strength and limited resistance to biodeterioration, restrict its direct application for exterior use. Therefore, appropriate modification techniques are required to enhance its performance.

Furfurylation is a chemical modification technique that is premised on impregnation and in-situ polymerization of furfuryl alcohol (FA), which provides enhanced resistance to moisture and biological degradation and enhanced dimensional stability and mechanical strength. In addition to extending a material’s service life, furfurylation also improves the physical and aesthetic quality of lignocellulosic materials. Previous studies have confirmed the effectiveness of furfurylation in improving wood’s durability and dimensional stability [4,5,6]. Although extensive research has been conducted on chemical modification methods including esterification [7] and polymer impregnation [2,8,9], only a limited number of techniques, namely acetylation, furfurylation, and DMDHEU treatment, have reached industrial implementation [10].

However, furfurylation is not the sole method that can be used to guarantee the best performance of particleboard products. The nature of adhesive applied is still a major consideration of what determines inter-particle bonding and durability in the long run. Practically, urea formaldehyde (UF), melamine formaldehyde (MF) and phenol formaldehyde (PF) are the most frequently used adhesives, each with its particular strength and restrictions according to the conditions of service and end-use needs. Previous studies have reported that furfurylated wood exhibits enhanced physical properties and improved delamination resistance, particularly when bonded with polyurethane and epoxy adhesives [11]. Nevertheless, limited information regarding the performance of furfurylated rattan-based particleboard bonded with conventional UF, MF, and PF adhesives is available.

Given the increasing interest in renewable modification agents, agricultural residue-based furfuryl alcohol has become one of the appealing and sustainable modifiers of composite materials. The previous successful application of furfurylation in plywood production [11] provides a scientific basis for extending this technology to particleboard manufacturing. Given the strategic importance of particleboard as a major composite product in Indonesia, the development of high-performance particleboard from furfurylated rattan waste is of considerable relevance. Therefore, this study aims to synthesize and characterize particleboard manufactured from furfurylated rattan stem waste using different adhesive systems (UF, MF, and PF), with a focus on improving its physical and mechanical properties. The novelty of this work lies in the integration of furfurylation treatment with conventional thermosetting adhesives in rattan particleboard production, providing new insights into the interaction between modified lignocellulosic materials and commercial resin systems. In addition, this study contributes to the development of value-added composite materials from underutilized rattan waste, supporting sustainable material innovation.

## 2. Materials and Methods

### 2.1. Materials

The materials used in this study included rattan stem waste obtained from HIMKI DPD Cirebon Raya, Cirebon, West Java, Indonesia. Furfuryl alcohol (FA, 97.7% purity) was employed as the modifying agent, while citric acid (99%) served as the catalyst. Both chemicals were purchased from Sigma Aldrich, Singapore. In the fabrication of particleboards, three types of commercial adhesives were utilized, namely phenol formaldehyde (PF, solid content 43.4%), melamine formaldehyde (MF, 54.0%), and urea formaldehyde (UF, 50.0%). These adhesives were obtained from PT. Palmolite Adhesive Industry (Probolinggo, East Java, Indonesia), and were applied without any additional purification.

### 2.2. Preparation of Rattan Particles

Waste Rattan stems were washed, then sliced and flaked in a dish-flaker machine. The moisture content of the obtained flakes was 12.30 at the start. The dimensions were between 20–50 mm in length and 2–5 mm in width. Using sieve classification, the particles were passed through a 5 mm sieve (U.S. no. 4) and retained on a sieve 2 mm (U.S. no. 10). Then, the flakes were subsequently dried in an oven at 60 °C until the moisture content was reduced to less than 8%.

### 2.3. Furfurylation Treatment

Rattan particles were immersed in an FA 50% aqueous solution (containing 3% of citric acid) for 24 h at room temperature to allow sufficient impregnation. After treatment, the particles were drained. The impregnated particles were subsequently dried and polymerized in an oven at 103 °C for 24–48 h. Untreated rattan particles were also prepared as control samples. From this treatment, the recorded weight percent gain was 43.3 ± 3.6% (*n* = 3).

### 2.4. Particleboard Production

PF, MF, and UF adhesives were used to produce particleboards with a 12% resin content, using the oven-dry weight of the particles. The glue was sprayed onto the particles and then mixed by hand in a glue mixer container to get a homogeneous distribution. The mat was then formed in a mold with dimensions of 300 mm × 300 mm × 10 mm and hot-pressed at 135 °C for PF-bonded boards and 120 °C for MF- and UF-bonded boards. The pressing time (10 min) and specific pressure (25 kg/cm^2^) were kept constant for all board types. After pressing, the boards were cooled at room temperature and conditioned for two weeks before characterizations.

### 2.5. Characterization

In this study, three particleboards were produced for each variant/treatment. For the characterization, test specimens were taken from all three boards and used as replicates for each variant/treatment. Table 1 shows a summary of parameter tested, sample types, specimen dimension, type of adhesive, and the applied standard/method.

#### 2.5.1. Physical Properties

##### Moisture Content

The oven-drying procedure was used as specified in JIS A 5908:2003 [12] to determine the moisture content of the particleboards [12]. Five (*n* = 5) test specimens 50 mm × 50 mm × 9 mm were randomly selected on each board. All the samples were weighed at first (W_1_) then dried in the oven at a temperature of 103 ± 2 °C until they reached a constant weight. The weight of the oven-dried substance (W_0_) was then recorded after drying in a desiccator. The following equation was used to determine the moisture content:

(1) $$\mathrm{MC}\left(\%\right)\;=\;\frac{W_{1}-\;W_{0}}{W_{0}}\;\times \;100$$

All measurements were conducted with five replications for each treatment, and the results were expressed as the mean values. The testing procedure followed JIS A 5908:2003 [12], which has also been applied in previous studies by Syukur et al. [13].

##### Density

The density of the particleboards was measured according to JIS A 5908:2003 [12]. Specimens (*n* = 5) with dimensions of 50 mm × 50 mm × 9 mm were cut from each board after a two-week conditioning period. The density (ρ) was calculated by dividing the oven-dried mass (M) of each specimen by its volume (V) using the following equation:

(2) $$\rho \;\left(\mathrm{kg}/m^{3}\right)\;=\;\frac{M}{V}$$

where M refers to the oven-dried weight of the specimen (kg) and V represents its volume (m^3^) as obtained from its measured length, width, and thickness. Five replications were used for each treatment.

##### Color Characteristics

The surface color of the particleboards was determined using the CIELab method by measuring L* (lightness), a* (red to green), and b* (blue to yellow) values, with a scanner (CanoScan 4400F, Canon, Tokyo, Japan) and Adobe Photoshop CS5. The color change (ΔE) of the specimens (*n* = 10) was also calculated based on the CIELab method, while classification followed Hunter Lab [14] and Hadi et al. [15]. The color parameters were expressed in the CIE Lab system*, where L represents lightness (0 = black, 100 = white), a indicates the green–red axis (ranging from −80 to +80), and b represents the blue–yellow axis (ranging from −70 to +70). Also, the chroma coordinate (C*) and hue coordinate (H*) were determined. Three points on the surface of each specimen were measured and the average values were taken. The overall color difference (ΔE) of the untreated and modified particleboards was calculated based on the following formula:

(3) $$∆E^{*}=\sqrt{{\left(∆L^{*}\right)}^{2}+{\left(∆a^{*}\right)}^{2}+{\left(∆b^{*}\right)}^{2}}.$$

This method is widely used for assessing the color changes in wood and lignocellulosic composites after chemical modification or thermal treatment [16].

##### Thickness Swelling

The thickness swelling (TS) of the particleboards was measured according to JIS A 5908:2003 [12]. Five specimens from each treatment with dimensions of 50 mm × 50 mm × 10 mm were prepared then dried to a constant weight and thickness. Then, the specimens were immersed in distilled water at 20 ± 2 °C for 2, 6, 24, 48, and 168 h. The specimens’ thickness was measured at the center of each panel using a digital caliper (±0.01 mm) before immersion (T_0_) and immediately after each immersion period (T_1_). The percentage of thickness swelling was calculated using the following equation:

(4) $$\mathrm{TS}\;\left(\%\right)\;=\;\frac{T_{1}-\;T_{0}}{T_{0}}\;\times 100.$$

##### Water Absorption

The water absorption of particleboard was determined simultaneously with the thickness swelling test according to JIS A 5908:2003 [12]. Five specimens from each treatment with dimensions of 50 mm × 50 mm × 10 mm were oven-dried to a constant weight (W_0_) prior to testing. The specimens were subsequently immersed in distilled water at 20 ± 2 °C for 2, 6, 24, 48, and 168 h. After each immersion interval, excess water on the surface was drained and the wet weight (W_1_) was recorded for each duration. The percentage of water absorption was calculated using the following formula:

(5) $$\mathrm{WA}\left(\%\right)\;=\;\frac{W_{1}-W_{0}}{W_{0}}\;\times \;100.$$

##### Leaching Value

The leaching value of the particleboard was performed at the same time as the thickness swelling test as per JIS A 5908:2003 [12]. Each of the treatments was tested with five specimens with dimensions of 50 mm × 50 mm × 10 mm that had been dried in the oven to a constant weight (W_0_). The samples were then placed into distilled water at 20 °C and allowed to stay there for 168 h. After the soaking period, any remaining water on the specimens’ surfaces was thoroughly removed and the specimens were dried at 103 °C over a period of 48 h and weighed (W_1_). The following formula was used to determine the leaching value of the specimens during immersion.

(6) $$\mathrm{Leaching}\left(\%\right)=\frac{W_{0}-W_{1}}{W_{0}}\;\times \;100.$$

#### 2.5.2. Mechanical Properties

##### Modulus of Elasticity and Modulus of Rupture

The modulus elasticity (MOE) and modulus of rupture (MOR) as part of the bending properties of the particleboards were measured according to JIS A 5908:2003 [12] using a universal testing machine (INSTRON 5989, Norwood, MA, USA). The six test specimens of each treatment were made with dimensions of 200 mm × 50 mm × 10 mm (length × width × thickness) and allowed two weeks to condition before the test. Bending test: The test was a one-point loading configuration test with a span length of 150 mm at 10 mm/min loading speed. The load and deflection data were automatically recorded while the test was underway. The data were then used to determine the MOE and MOR values using the standard equations.

##### Internal Bonding

The internal bonding (IB) strength of the particleboards was calculated in accordance with JIS A 5908:2003 [12] on a universal testing machine (INSTRON 5989, USA). The conditioned boards were cut into test specimens (*n* = 6) 50 mm × 50 mm × 9 mm and bonded at the center to loading blocks using epoxy (Ar-aldhite) adhesive. This was followed by a tensile load that was applied at right angles to the board surface at a speed of the crosshead of 2 mm/min until failure. The highest load (Fmax) at failure was measured and then divided by the cross-sectional area (A) of the specimen to get the strength of the IB in N/mm^2^. Each treatment was tested using one specimen with six replications, and the mean values were reported. This procedure is in line with the particleboard testing procedures employed in earlier studies [17].

(7) $$\mathrm{IB}\;(N/{mm}^{2})=\frac{Fmax}{A}\;$$

##### Hardness

Hardness was evaluated following the ASTM D143-22 standard using the Janka method [18]. Specimens measuring approximately 100 mm × 50 mm × board thickness were prepared and conditioned at 20 ± 2 °C and 65 ± 5% relative humidity until they reached a stable mass. The test was carried out using a universal testing machine (INSTRON 5989, USA) equipped with a steel ball indenter 11.28 mm in diameter. During testing, each specimen was positioned on a rigid base and the indenter was applied perpendicular to the surface at a minimum distance of 20 mm from the edges. The load was applied continuously at a constant crosshead speed of 6 mm min^−1^ until the indenter penetrated to half of its diameter (5.64 mm). The maximum load recorded at this penetration depth was taken as the Janka hardness value. A minimum of six specimens (*n* = 6) were tested for each board type, and the results were expressed as the mean value along with the standard deviation.

Janka Hardness (N) = Fmax(8)

##### Screw Holding Strength

The screw holding strength (SHP) of the particleboards was measured in accordance with JIS A 5908:2003 [12] with the help of a universal testing machine (INSTRON 5989, USA). Conditioned boards with the following dimensions: 100 mm × 50 mm × 9 mm were prepared as test specimens (*n* = 6). Wood screws (3.5 mm round × 30 mm long) were put into the specimens vertically at a screw depth of 15 mm at two points per sample. The screws were then pulled out at a crosshead speed of 2 mm/min and the maximum withdrawal load was taken. The values of SHP were given in Newtons (N).

### 2.6. Data Analysis

A randomized complete block design (RCBD) was used to evaluate the effects of the treatments on all response variables for data analysis. The rattan condition, which had untreated and furfurylated rattan, was the first factor. The second factor was adhesive type, i.e., PF, MF, and UF. Further analysis was done through Duncan analysis in case a factor was significantly different at *p* ≤ 0.05. The statistical tests and data processing were done in Microsoft Excel and IBM SPSS Statistics version 27.

## 3. Results and Discussion

### 3.1. Physical Properties

**Figure 1:**
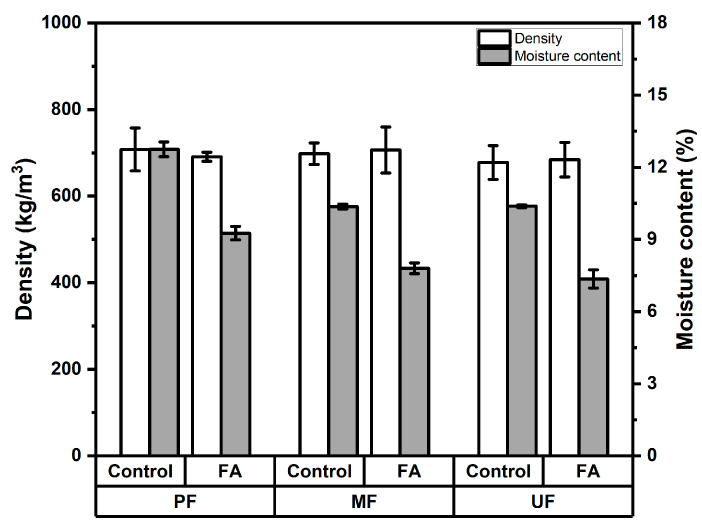
Density and moisture content of rattan particleboard.

The density of rattan particleboards manufactured using three different adhesives (PF, MF, and UF), ranged from 680 to 710 kg/m^3^ for both control and furfurylated rattan particles. As presented in Figure 1, particleboards bonded with PF adhesive showed a slight reduction in density, decreasing from 710 kg/m^3^ in the control boards to 690 kg/m^3^ after furfurylation. In contrast, boards produced using MF adhesive exhibited a marginal increase, with density rising from 0.70 g/cm^3^ in the control condition to 0.71 g/cm^3^ in the furfurylated samples. Meanwhile, boards bonded with UF adhesive showed identical density values of 680 kg/m^3^ for both treatments. These results indicate that the furfurylation treatment did not significantly affect the density of the produced particleboards, as revealed in Table 2. The relatively uniform density among all samples suggests that board density was primarily influenced by the manufacturing parameters, including mat formation, pressing condition (time, temperature, and pressure), the predetermined target density, and the chemical modification of the rattan particles or the adhesive type used.

Furfurylation alters the structure of the cell wall through the in situ polymerization of furfuryl alcohol within the lignocellulosic matrix. As a result, the mass of individual particles may increase due to the formation of polyfurfuryl alcohol within both the cell walls and lumens [19,20]. This modification takes place when furfuryl alcohol diffuses into the cell walls and undergoes in situ polymerization, leading to cell wall bulking and an increase in the overall mass of the treated material [21,22]. However, in particleboard manufacturing, the compaction during hot pressing tends to equalize the board’s density, thereby minimizing the influence of such modifications on the final panel’s density. Additionally, the similar density values across different adhesive systems (Table 2) indicate that these adhesives did not significantly alter the compaction behavior of the rattan particles during board formation. These results are in line with further Duncan analysis, as shown in Table 3. Therefore, the density results confirm that the targeted board density was successfully achieved according to the JIS A 5908-2003 [12] (400–900 kg/m^3^) and that the furfurylation treatment did not adversely affect the physical consolidation of the rattan particleboards.

In terms of moisture content of rattan particleboards, Figure 1 shows a clear reduction after furfurylation treatment. The moisture content of PF-bonded boards decreased from 12.75% in the control samples to 9.26% in the furfurylated samples. Similarly, MF-bonded boards exhibited a reduction from 10.37% (control) to 7.80% (furfurylated) and UF-bonded boards showed a decrease from 10.38% to 7.36% after treatment. These results indicate that furfurylation effectively reduced the hygroscopic nature of the rattan particles, resulting in lower moisture content in the produced particleboards. The variance analysis shown in Table 2 reveals that the adhesive type and treatment process used in rattan significantly affected to moisture content. The Duncan analysis shown in Table 3 indicates that the UF-bonded board with furfrylated rattan showed the lowest moisture content. The reduction in moisture content can be attributed to the polymerization of furfuryl alcohol within the cell wall structure, which reduces the availability of hydroxyl groups responsible for moisture absorption [22,23]. During furfurylation, furfuryl alcohol penetrates the lignocellulosic structure and polymerizes in situ, forming polyfurfuryl alcohol within the cell walls and lumens, which decreases hygroscopicity and moisture uptake [20,24]. Consequently, the furfurylated rattan particles exhibit improved resistance to moisture uptake. In addition, the consistent reduction observed across all adhesive systems suggests that the chemical modification plays a more dominant role in controlling moisture content than the type of adhesive used during particleboard manufacturing. The enhanced resistance to moisture is expected to contribute to improved dimensional stability and long-term durability of rattan particleboards. This can be attributed to the effect of furfurylation, which has been shown to reduce water uptake and improve the stability of lignocellulosic materials [25,26]. All of the manufactured boards meet JIS standards (5–13%) except for the control particleboards produced using PF adhesive.

**Figure 2:**
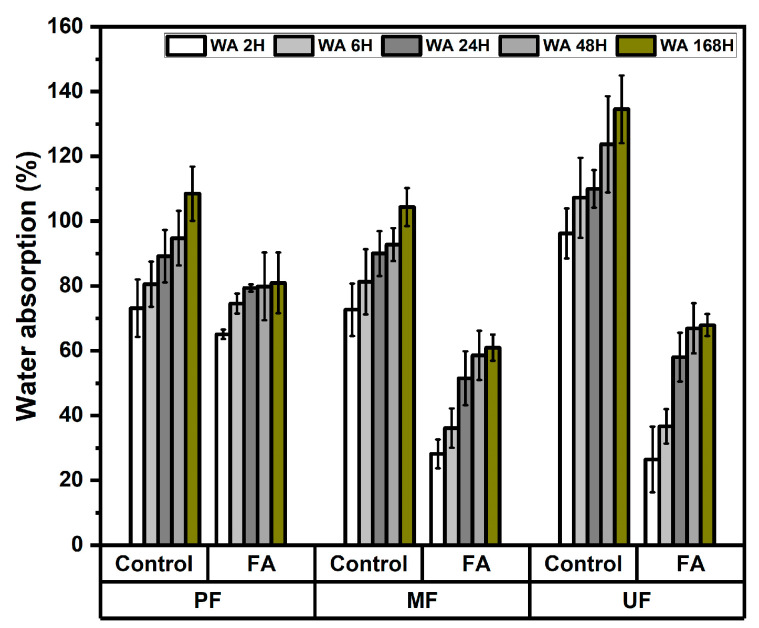
Water absorption content of rattan particleboard.

The water absorption behavior of rattan particleboards was evaluated at immersion times of 2, 6, 24, 48, and 168 h. The result presented in Figure 2 shows that water absorption increased with longer immersion time for all samples. However, particleboards produced from furfurylated rattan exhibited significantly lower water absorption compared to the control boards. For PF-bonded boards, water absorption decreased from 73.12–108.5% in the control samples to 65.09–80.97% in the furfurylated samples. A more pronounced reduction was observed in MF-bonded boards, where the values decreased from 72.66–104.37% to 28.19–60.96% after treatment. Similarly, the UF-bonded boards showed a substantial reduction from 96.23–134.57% (control) to 26.43–67.94% (furfurylated). The variance analysis shown in Table 2 reveals that the furfurylation treatment had a highly significant effect on water absorption at all immersion times, whereas the adhesive type showed no significant effect up to 48 h but became significant at 168 h. The Duncan’s multiple range test shown in Table 3 further confirms that treatment had a strong influence on water absorption, with furfurylated boards consistently showing significantly lower values than control boards. This demonstrates that furfurylation effectively improves the hydrophobic properties and dimensional stability of rattan particleboards. Previous studies reported that furfurylation significantly reduces the water absorption and swelling of lignocellulosic materials by modifying the wood cell wall and improving resistance to moisture penetration [23,24,27,28]. Consequently, the furfurylated rattan particles exhibit improved resistance to water penetration and reduced hygroscopicity due to the formation of polyfurfuryl alcohol within the lignocellulosic matrix [22]. As a result, the dimensional stability of the resulting particleboards is enhanced, leading to improved performance under prolonged water exposure [29].

**Figure 3:**
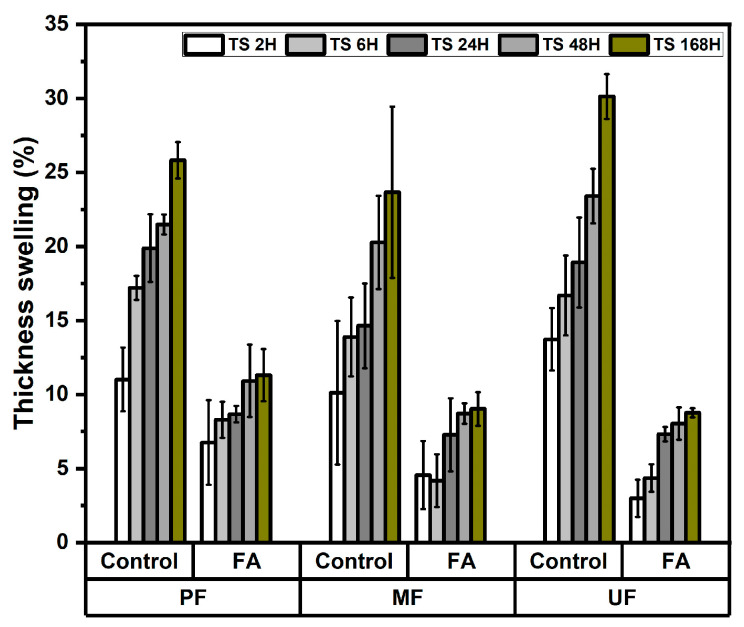
Thickness swelling of rattan particleboard.

The thickness swelling behavior of rattan particleboards was evaluated after immersion in water for 2, 6, 24, 48, and 168 h. Figure 3 shows that the thickness swelling increased with longer immersion time for all particleboards. However, boards manufactured from furfurylated rattan particles exhibited substantially lower swelling values compared to the control boards. This phenomenon was similar with regard to water absorption. In the PF-bonded boards, thickness swelling decreased from 11.02–25.82% in control samples to only 6.76–11.30% in furfurylated boards. Similarly, MF-bonded boards showed a reduction from 10.13–23.65% to 4.56–9.03% after treatment. The most significant improvement was observed in UF-bonded boards, where thickness swelling decreased from 13.73–30.13% (control) to 2.99–8.77% (furfurylated). The variance analysis shown in Table 2 indicates that the furfurylation treatment had a highly significant effect on thickness swelling at all immersion times, whereas the adhesive type showed limited influence, being significant only at 6 h immersion. The Duncan’s multiple range test shown in Table 3 also confirms that the furfurylated boards had significantly lower thickness swelling values compared to the control boards. This result demonstrates that furfurylation effectively enhances the dimensional stability and water resistance of rattan particleboards. Previous studies reported that furfurylation improves dimensional stability and significantly reduces swelling in lignocellulosic materials by modifying the internal structure of the cell wall [23,27]. The reduction in thickness swelling can also be attributed to the polymerization of furfuryl alcohol within the lignocellulosic cell wall, which reduces the availability of hydroxyl groups and blocks micro voids that facilitate water penetration [24,28]. In the furfurylation process, furfuryl alcohol diffuses into the cell wall and subsequently polymerizes in situ, leading to the formation of polyfurfuryl alcohol. This results in cell wall bulking and reduces the accessibility of moisture within the material [20,24]. Consequently, the furfurylated rattan particles exhibit improved resistance to water uptake, leading to enhanced dimensional stability of the resulting particleboards [29]. All furfurylated rattan particleboards met the JIS standard requirement (maximum 12% thickness swelling), except for the control particleboards for each type of adhesive.

**Figure 4:**
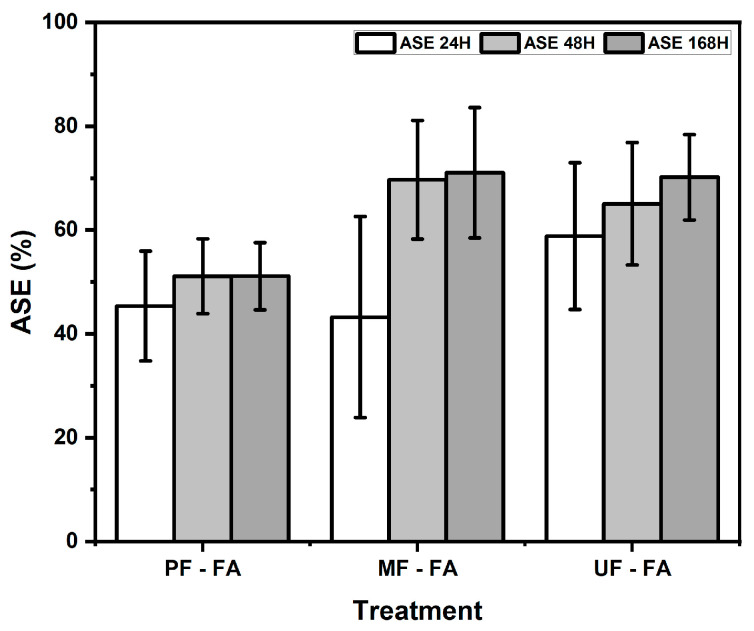
Anti-swelling of rattan particleboard.

The anti-swelling efficiency (ASE) of the rattan particleboards was evaluated after 24, 48, and 168 h of water immersion. Figure 4 shows that furfurylation significantly improved the dimensional stability of the rattan particleboards, as reflected by ASE values ranging from 43.25% to 71.06%. For PF-bonded boards, the ASE values increased from 45.37% at 24 h to approximately 51% at longer immersion times. In MF-bonded boards, the ASE increased substantially, from 43.25% to 69.68% and 71.06% at 48 h and 168 h, respectively. Similarly, UF-bonded boards showed ASE values of 58.83%, 65.08%, and 70.18% at 24, 48, and 168 h. However, variance analysis indicated that the adhesive type used had no significant effect on ASE values, suggesting that the improvement in dimensional stability was mainly attributed to the furfurylation treatment. The Duncan’s multiple range test (Table 3) indicated no significant differences in ASE values among particleboards bonded with PF, MF, and UF adhesives. This result suggests that the furfurylation treatment consistently improved the dimensional stability of rattan particleboards, regardless of the adhesive system used. The enhanced ASE values can also be explained by the polymerization of furfuryl alcohol within the lignocellulosic structure of rattan, which has a similar effect to that observed in water absorption. During the furfurylation process, furfuryl alcohol penetrates into the cell wall and polymerizes in situ to form polyfurfuryl alcohol, which bulks the cell wall structure and reduces the hygroscopicity of the material [20,30]. As a result, the treated particles exhibit reduced swelling behavior and improved resistance to moisture-induced dimensional changes [23,29]. Consequently, the treated particles exhibited reduced swelling behavior, resulting in improved dimensional stability of the produced particleboards.

**Figure 5:**
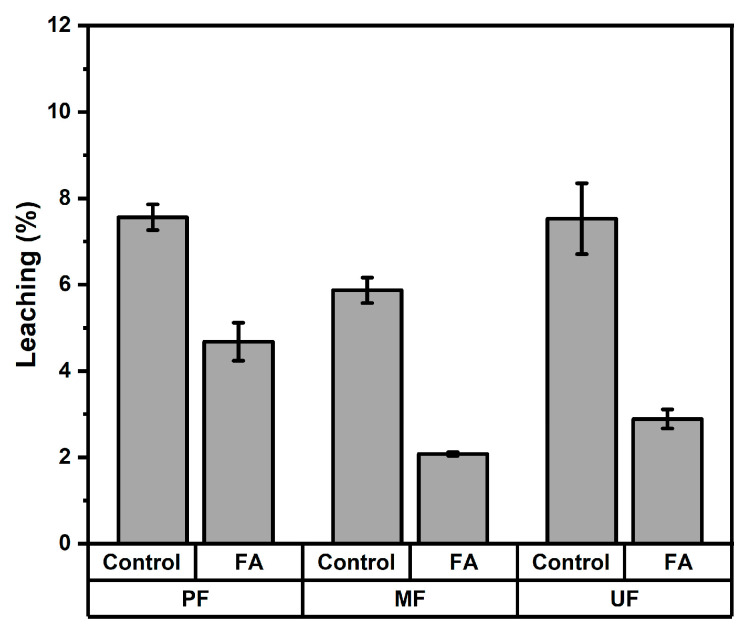
Leaching test of rattan particleboard.

The leaching behavior of rattan particleboards was evaluated to determine the stability of board components during water exposure. The leaching values were measured after 168 h of immersion. The results are shown in Figure 5, presenting the conclusion that particleboards produced from furfurylated rattan exhibited significantly lower leaching values than the control boards. For PF-bonded boards, the leaching value decreased from 7.56% in control samples to 4.68% in furfurylated boards. Similarly, MF-bonded boards showed a reduction from 5.87% to 2.08%, while UF-bonded boards decreased from 7.53% to 2.89% after treatment. The variance analysis shown in Table 2 indicates that both the adhesive type and furfurylation treatment had highly significant effects on leaching values. The Duncan’s multiple range test in Table 3 also confirmed significant differences among adhesive types. Furthermore, the treatment comparison showed that the furfurylated boards (3.21%) had substantially lower leaching values than the control boards (6.98%), indicating that furfurylation effectively improved the resistance of the rattan particleboards to component loss during water exposure. The reduced leaching observed in the furfurylated boards can be attributed to the polymerization of furfuryl alcohol within the lignocellulosic matrix of rattan, which forms a more stable and less water-soluble structure [20,30]. In the furfurylation process, furfuryl alcohol diffuses into the cell wall and subsequently polymerizes in situ to form polyfurfuryl alcohol. The resulting polymer can interact with lignin and hemicellulose, allowing it to become fixed within the wood structure [31]. This polymer network reduces the dissolution and migration of extractives and other soluble components during water immersion, thereby improving the leaching resistance of the material [32,33]. Consequently, the furfurylation treatment enhances the chemical stability and durability of rattan particleboards when exposed to moisture. In addition, there is a possibility that a reaction occurred between the adhesives and the polyfurfuryl alcohol in the wood structure, forming a complex polymer that improved the performance of the corresponding particleboards, including their resistance to leaching.

**Figure 6:**
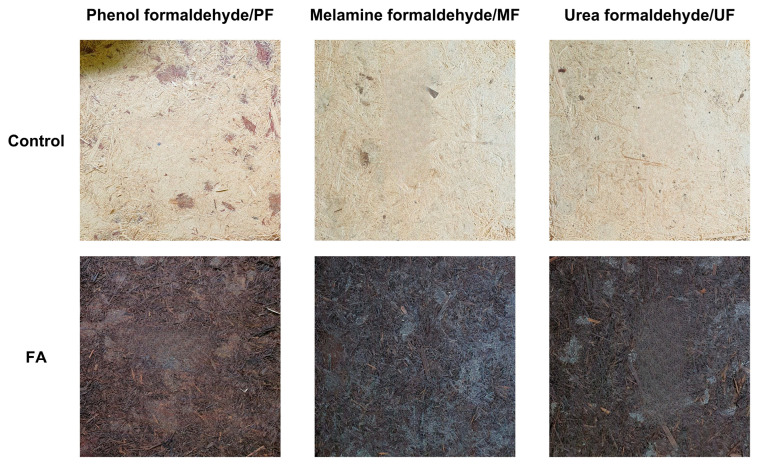
Appearance of rattan particleboard modified with different treatment.

The visual appearance of the boards can be observed in Figure 6 and their color characteristics are summarized in Table 4. The parameters include lightness (L*), red–green coordinate (a*), yellow–blue coordinate (b*), chroma (C*), hue angle (H*), and color difference (ΔE). The results showed that furfurylation significantly affected the color properties of the boards. The lightness (L*) values decreased noticeably after furfurylation, from 79.07–86.87 in control boards to approximately 36.73–37.20 in furfurylated boards, indicating substantial darkening of the material. Similar observations have been reported in previous studies, where furfurylated wood exhibited a darker color compared with untreated wood due to chemical reactions occurring during the modification process [27,34]. This color change is associated with the polymerization reactions of furfuryl alcohol during the curing process, which produce dark-colored polymeric compounds within the lignocellulosic matrix [19,25]. The a* values showed a slight increase in some adhesive systems, indicating a shift toward redder tones, while the b* values varied depending on adhesive type. The C* values, which represent color intensity or saturation, tended to decrease in PF and UF boards after furfurylation, indicating slightly less vivid colors. In contrast, MF boards showed a slight increase in chroma after treatment. The H* values also changed considerably, reflecting a shift in the dominant color tone of the boards after modification. The total color difference (ΔE) values ranged from 42.12 to 50.54, demonstrating a pronounced visual difference between untreated and furfurylated boards. The variance analysis shown in Table 2 reveals that both the adhesive type and furfurylation treatment significantly affected most color parameters, although the adhesive type had no significant effect on lightness. The Duncan’s multiple range test shown in Table 3 confirmed significant differences among adhesive types and treatments, demonstrating that furfurylation was the primary factor influencing the color characteristics of the rattan particleboards. Overall, the results suggest that furfurylation substantially modifies the visual appearance of rattan particleboards, producing darker and more uniform color characteristics due to the formation of polymerized furfuryl alcohol within the cell wall structure [35].

### 3.2. Mechanical Properties

**Figure 7:**
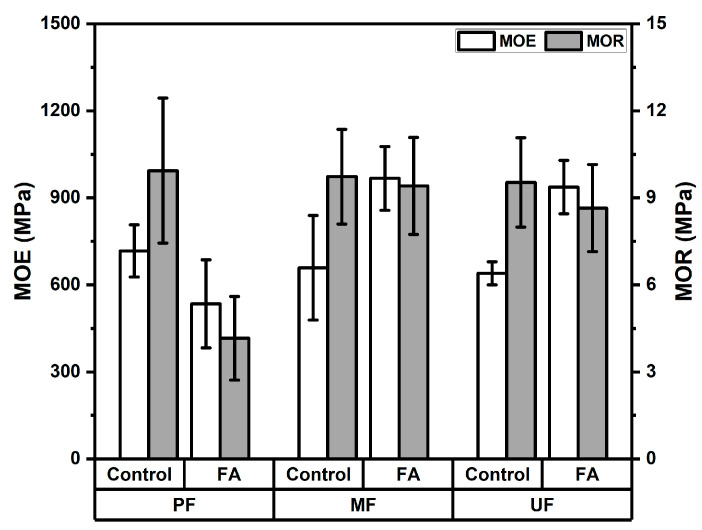
Modulus of elasticity and modulus of rupture rattan particleboard.

The modulus of elasticity (MOE) of rattan particleboards ranged from 535 to 967 MPa, as shown in Figure 7. In PF-bonded boards, the MOE decreased from 717 MPa in the control boards to 535 MPa in the furfurylated boards. In contrast, boards bonded with MF adhesive showed a substantial increase in MOE from 659 MPa to 967 MPa after furfurylation. Similarly, UF-bonded boards exhibited an increase from 640 MPa to 937 MPa following treatment. However, the variance analysis shown in Table 5 shows that neither adhesive type nor furfurylation treatment had a significant effect on the MOE values. The Duncan’s multiple range test shown in Table 6 indicates that MF-bonded boards had the highest average MOE (812 MPa), followed by UF (788 MPa) and PF (626 MPa). Although furfurylated boards exhibited a higher average MOE (813 MPa) compared to the control boards (671 MPa), the difference was not statistically significant.

Compared with other lignocellulosic particleboards reported in the literature, the MOE values obtained in this study are relatively lower. For example, bamboo-based particleboards have been reported to reach MOE values of approximately 1958 MPa or higher depending on particle size and board composition [36], while hybrid wood–bamboo particleboards can reach values of about 2466–2922 MPa [37]. Similarly, particleboards manufactured from bamboo veneer waste have shown MOE values of approximately 2650 MPa [38]. As a result, none of the particleboards produced in this study satisfied the JIS A 5908 requirement for MOE (minimum 2000 MPa).

The relatively low MOE values observed in the present study may be attributed to several factors, including particle geometry, board density, adhesive distribution, and internal bonding quality. Previous studies have reported that board density and particle size strongly influence bending stiffness, with higher densities and finer particles generally producing boards with greater MOE values due to improved inter-particle bonding and stress transfer [36,39]. In addition, the relatively low stiffness of rattan particles and possible incomplete consolidation during board pressing may have limited the development of bending strength in the boards.

The increase in MOE observed in some treatments may also be associated with the polymerization of furfuryl alcohol within the lignocellulosic structure of rattan, which can increase cell wall rigidity and enhance the stiffness of lignocellulosic materials [19]. Nevertheless, the results suggest that the overall stiffness of rattan particleboards in this study was not significantly influenced by the furfurylation treatment or adhesive type, and further optimization of board density, particle geometry, and adhesive formulation may be necessary to achieve the MOE values required by the JIS standard.

The modulus of rupture (MOR) values of the rattan particleboards, as presented in Figure 7, ranged from 4.16 to 9.94 MPa. The MOR value of PF-bonded boards decreased noticeably from 9.94 MPa in the control boards to 4.16 MPa in the furfurylated boards, indicating a reduction in bending strength after treatment. In contrast, MF-bonded boards showed only a slight decrease from 9.73 MPa to 9.41 MPa, while UF-bonded boards decreased moderately from 9.53 MPa to 8.65 MPa following furfurylation. The variance analysis shown in Table 5 indicates that adhesive type had no significant effect on MOR values, whereas the furfurylation treatment had a highly significant effect. The Duncan’s multiple range test shown in Table 6 indicates that MF-bonded boards exhibited the highest average MOR value (9.56 MPa), followed by UF (9.09 MPa) and PF (7.04 MPa). Additionally, the control boards had higher MOR values (9.73 MPa) than the furfurylated boards (7.40 MPa). Compared with other lignocellulosic particleboards, the MOR values obtained in this study fall within the lower range reported for non-wood particleboards. For instance, bamboo-based particleboards have been reported to exhibit MOR values of approximately 15 MPa or higher depending on particle composition and manufacturing parameters [38]. Similarly, hybrid wood–bamboo particleboards have shown bending strength values of about 15–18 MPa, indicating stronger structural performance compared with boards produced solely from certain lignocellulosic residues [37]. The observed decrease in bending strength following furfurylation can be associated with the in situ polymerization of furfuryl alcohol within the cell wall. Although this modification enhances stiffness, it may also induce brittleness and reduce the effectiveness of interfacial bonding between particles and the adhesive. Previous studies have reported that furfurylation modifies the cell wall through in-situ polymerization of furfuryl alcohol, which can alter the mechanical behavior of lignocellulosic materials by increasing rigidity and sometimes reducing flexibility under bending loads [19,40]. In addition, the bending strength of particleboards is strongly influenced by board density, particle geometry, and bonding quality between particles and adhesives [39]. Although a reduction in MOR was observed in some treatments, most of the boards produced in this study satisfied the JIS A 5908 requirement (minimum 8 MPa). An exception was found in the furfurylated rattan particleboards bonded with PF adhesive. The relatively lower mechanical performance observed in PF-bonded boards after furfurylation may be explained by several factors. Besides the deposition of polyfurfuryl alcohol within the cell lumens, which may reduce adhesive penetration (thereby limiting effective mechanical interlocking) and decrease the availability of accessible hydroxyl groups (thus weakening chemical bonding between the adhesive and the substrate), the formation of a more rigid polymer network may increase brittleness, negatively affecting bending strength. These combined effects likely contribute to the reduced performance observed in PF systems compared to MF and UF adhesives.

**Figure 8:**
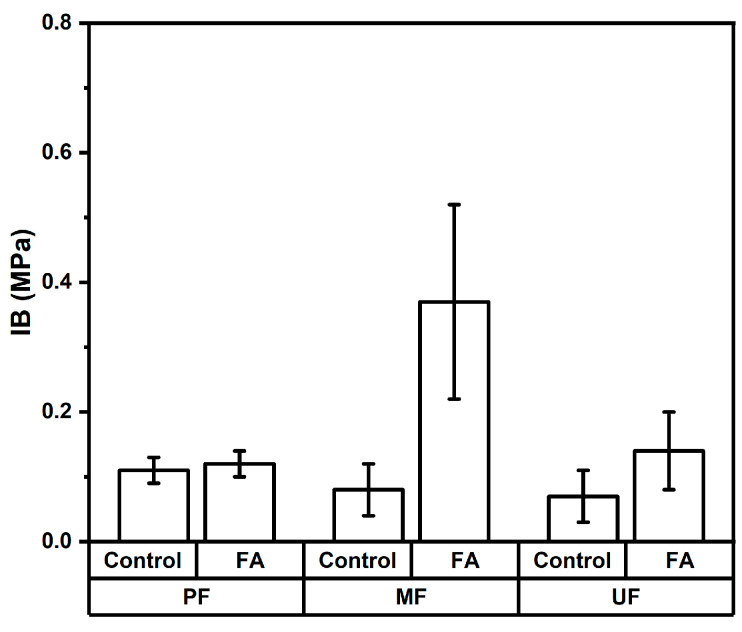
Internal bonding of rattan particleboard.

The internal bonding (IB) strength of rattan particleboard is shown in Figure 8. The IB strength of rattan particleboards ranged from 0.07 to 0.37 MPa. The IB value of PF-bonded boards slightly increased from 0.11 MPa in the control boards to 0.12 MPa in the furfurylated boards. A substantial improvement was observed in MF-bonded boards, where IB increased from 0.08 MPa to 0.37 MPa after furfurylation. Similarly, UF-bonded boards showed an increase from 0.07 MPa in control samples to 0.14 MPa in furfurylated boards. The variance analysis shown in Table 5 shows that adhesive type had no significant effect on IB values, whereas the furfurylation treatment had a highly significant effect. The Duncan’s multiple range test shown in Table 6 confirms that the average IB value of furfurylated boards (0.21 MPa) was higher than that of the control boards (0.08 MPa). The improvement in internal bonding after furfurylation may be attributed to the polymerization of furfuryl alcohol within the lignocellulosic structure of rattan, which can enhance particle surface stability and promote better interaction between the rattan particles and the adhesive matrix. The formation of polymerized furfuryl alcohol within the cell wall can modify the surface chemistry and improve the compatibility between lignocellulosic particles and adhesives during board consolidation. Similar improvements in the bonding performance of modified lignocellulosic composites have been reported in previous studies [41]. The internal bonding strength of particleboards is affected by several factors, including particle geometry, board density, adhesive content, and the quality of interfacial bonding between the particles and the resin [42]. Studies of particleboards manufactured from various lignocellulosic residues have reported IB values ranging from approximately 0.03 to 0.69 MPa depending on the raw material composition and manufacturing parameters [43]. Consequently, the modified particles contribute to stronger inter-particle bonding within the particleboard structure. Among the treatments evaluated in this study, particleboard manufactured from furfurylated rattan using MF adhesive was the only board that met the JIS A 5908 standard requirement for internal bonding strength (minimum 0.15 MPa).

**Figure 9:**
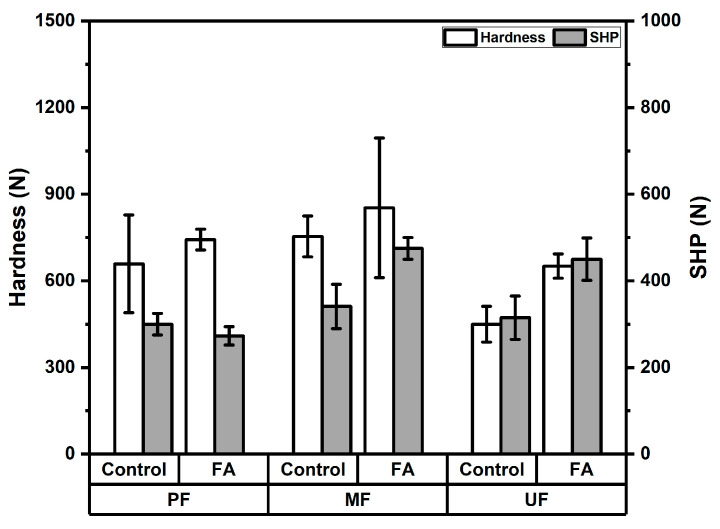
Hardness and screw holding power of rattan particleboard.

The hardness value of particleboards was showed in Figure 9. The hardness of rattan particleboards was ranged from 450 to 853 N. The hardness of PF-bonded boards increased from 659 N in the control boards to 743 N in the furfurylated boards, indicating improved resistance to surface indentation after treatment. Similarly, MF-bonded boards showed an increase from 754 N to 853 N, while UF-bonded boards exhibited a substantial increase from 450 N in the control boards to 651 N in the furfurylated boards following furfurylation. The variance analysis shown in Table 5 shows that both the adhesive type and furfurylation treatment had highly significant effects on hardness values. The Duncan’s multiple range test in Table 6 shows that MF-bonded boards had the highest average hardness value (7877 N), followed by PF (6876 N) and UF (5399 N). Furthermore, the average hardness of furfurylated boards (7345 N) was higher than that of control boards (6090 N). The improvement in hardness after furfurylation can be attributed to the polymerization of furfuryl alcohol within the cell wall structure of rattan, which increases the rigidity and density of the particle surface. In the furfurylation process, furfuryl alcohol diffuses into the cell wall and subsequently polymerizes in situ to form polyfurfuryl alcohol. This polymer contributes to strengthening the lignocellulosic matrix and improves its mechanical properties, particularly hardness and stiffness [19,44,45,46]. Previous studies have also reported that furfurylated wood exhibits increased hardness and surface durability due to the bulking effect of polymerized furfuryl alcohol within the cell wall structure [40,44,45,46]. In addition, the hardness of particleboards is influenced by board density, particle bonding quality, and surface consolidation during hot pressing [42,44,45,46,47]. The increased rigidity of the furfurylated rattan particles therefore contributes to improved resistance of the particleboard surface to indentation and mechanical wear.

The screw holding power of the rattan particleboards ranged from 273 to 475 N. The results are shown in Figure 9. The screw holding power of PF-bonded boards decreased slightly from 300 N in the control boards to 273 N in the furfurylated boards. In contrast, MF-bonded boards showed a substantial improvement from 341 N to 475 N after furfurylation. Similarly, UF-bonded boards exhibited an increase from 315 N in control samples to 450 N in furfurylated boards. The variance analysis shown in Table 5 shows that both the adhesive type and furfurylation treatment had highly significant effects on screw holding power. The Duncan’s multiple range test shown in Table 6 shows that MF-bonded boards had the highest average screw holding power (408 N), followed by UF (382 N) and PF (286 N). Furthermore, the average screw holding power of furfurylated boards (399 N) was higher than that of control boards (318 N). The improvement in screw holding performance after furfurylation may be attributed to the polymerization of furfuryl alcohol within the lignocellulosic matrix, which increases particle rigidity and enhances the internal structure of the board, thereby improving resistance to screw withdrawal forces. During the furfurylation process, furfuryl alcohol penetrates the cell wall and polymerizes to form polyfurfuryl alcohol, reinforcing the lignocellulosic structure and increasing the mechanical resistance of the material [19]. In addition, screw holding capacity in particleboards is strongly influenced by board density, internal bonding strength, and the quality of adhesion between particles and resin, which collectively determine the resistance of the board structure to withdrawal forces [42]. Studies on particleboards produced from lignocellulosic materials have reported screw holding values in the range of approximately 250–500 N depending on raw materials, adhesive systems, and board density [43,48,49]. Overall, the results indicate that furfurylation generally improved the screw holding performance of rattan particleboards. Most of the boards produced in this study met the JIS A 5908 requirement for screw holding power (minimum 300 N), with the exception of the furfurylated rattan particleboards bonded with PF adhesive.

Based on the improved dimensional stability, reduced water absorption, and enhancements of some mechanical performance parameters, the developed particleboards show strong potential for applications in interior environments with moderate to high humidity exposure, such as furniture components, wall paneling, and flooring substrates. The furfurylation treatment significantly improves resistance to moisture-related degradation; however, full exterior applications remain limited due to the lack of long-term weathering data and potential adhesive degradation under harsh environmental conditions. Therefore, while the treated boards exhibit enhanced performance, their use is currently more suitable for semi-exterior or protected applications rather than direct outdoor exposure.

## 4. Conclusions

This study demonstrates that furfurylation using furfuryl alcohol can be an effective modification approach to improving the dimensional stability of rattan-based particleboards bonded with phenol formaldehyde (PF), melamine formaldehyde (MF), and urea formaldehyde (UF) adhesives. While board density remained relatively constant (0.68–0.71 g cm^−3^), the treatment significantly reduced moisture content, water absorption, thickness swelling, and leaching. The relatively high anti-swelling efficiency values (43.25–71.06%) indicate improved resistance to moisture-induced degradation, which is likely associated with the polymerization of furfuryl alcohol within the lignocellulosic matrix and the resulting reduction in hydroxyl accessibility.

In terms of mechanical performance, furfurylation contributed to improvements in selected properties, including internal bonding strength, hardness, and screw holding power, suggesting enhanced inter-particle interactions and surface resistance. However, its effect on bending properties was limited, with little improvement in the modulus of elasticity and a reduction in the modulus of rupture observed in some cases. This may indicate increased rigidity accompanied by a tendency toward brittleness in the composite matrix. Among the adhesive systems, MF-bonded boards exhibited the most balanced overall performance. Despite these limitations, the developed particleboards show potential for non-structural and decorative applications. Additionally, furfurylation resulted in darker and more uniform surface characteristics, which may enhance aesthetic value.

It should be noted that formaldehyde emissions, as well as FTIR and morphological analyses, were not evaluated in this study and are therefore recognized as limitations. Although furfuryl alcohol may contribute to reduced emission pathways through polymer network formation, this assumption requires further verification. Future research should include formaldehyde emissions testing, along with FTIR, NMR, biostability, and morphological analyses, to better assess environmental safety, ensure compliance with relevant international standards, and improve our understanding of the interactions between furfurylated lignocellulosic materials and adhesive systems.

## Figures and Tables

**Table 1:** Summary of parameter tested, specimen dimension, the applied standard/method, sample types, and the type of adhesive.

Property/Test	Specimen Dimensions (mm) *	Standard/Method	Sample Types	Adhesive Systems
Moisture content	50 × 50 × 10	JIS A 5908:2003	Untreated PB, Furfurylated PB	PF, MF, UF
Density	50 × 50 × 10	JIS A 5908:2003	Untreated PB, Furfurylated PB	PF, MF, UF
Color characteristics	50 × 50 × 10	CIELab Method	Untreated PB, Furfurylated PB	PF, MF, UF
Thickness swelling	50 × 50 × 10	JIS A 5908:2003	Untreated PB, Furfurylated PB	PF, MF, UF
Water absorption	50 × 50 × 10	JIS A 5908:2003	Untreated PB, Furfurylated PB	PF, MF, UF
Leaching	50 × 50 × 10	JIS A 5908:2003	Untreated PB, Furfurylated PB	PF, MF, UF
MOE & MOR (Bending)	200 × 50 × 10	JIS A 5908:2003	Untreated PB, Furfurylated PB	PF, MF, UF
Internal bonding	50 × 50 × 10	JIS A 5908:2003	Untreated PB, Furfurylated PB	PF, MF, UF
Hardness (Janka)	100 × 50 × 10	ASTM D143-22	Untreated PB, Furfurylated PB	PF, MF, UF
Screw holding strength	100 × 50 × 10	JIS A 5908:2003	Untreated PB, Furfurylated PB	PF, MF, UF

Note: * In this study, three particleboards were produced for each variant. For the characterization, test specimens were taken from all three boards and used as replicates for each variant/treatment, rather than being obtained from a single particleboard. PB is particle board, PF is phenol formaldehyde, MF is melamine formaldehyde, UF is urea formaldehyde.

**Table 2:** Variance analysis of physical properties rattan particleboard.

Parameter	Adhesive Type			Treatment		
	F	df	*p*-Value	F	df Total	*p*-Value
Density	0.57	2	0.57 ns	0.00	1	1.00 ns
Moisture content	82.52	2	0.00 **	410.42	1	0.00 **
Water absorption 2 h	2.08	2	0.16 ns	29.49	1	0.00 **
Water absorption 6 h	2.00	2	0.17 ns	26.39	1	0.00 **
Water absorption 24 h	2.58	2	0.11 ns	36.25	1	0.00 **
Water absorption 48 h	3.39	2	0.63 ns	32.59	1	0.00 **
Water absorption 168 h	4.10	2	0.40 **	72.80	1	0.00 **
Thickness swelling 2 h	0.39	2	0.68 ns	22.69	1	0.00 **
Thickness swelling 6 h	5.81	2	0.01 **	132.76	1	0.00 **
Thickness swelling 24 h	3.17	2	0.07 ns	84.91	1	0.00 **
Thickness swelling 48 h	1.05	2	0.37 ns	158.78	1	0.00 **
Thickness swelling 168 h	1.66	2	0.22 ns	138.01	1	0.00 **
ASE 24 h	0.93	2	0.44 ns	-	-	-
ASE 48 h	2.62	2	0.15 ns	-	-	-
ASE 168 h	4.26	2	0.07 ns	-	-	-
Leaching	21.55	2	0.00 **	198.51	1	0.00 **
L*	2.70	2	0.07 ns	1072	1	0.00 **
a*	12.69	2	0.00 **	22.80	1	0.00 **
b*	20.65	2	0.00 **	56.77	1	0.00 **
C*	8.20	2	0.00 **	26.25	1	0.00 **
H*	33.67	2	0.00 **	80.33	1	0.00 **
∆E	4.25	2	0.02 **	-	-	-

Note: (**) indicates highly significant differences at *p* ≤ 0.01; (ns); not significant; (-) no data.

**Table 3:** Duncan’s multiple range test of physical properties rattan particleboard.

Parameter	Adhesive Type			Treatment	
	PF	MF	UF	Control	FA
Density	698 a	701 a	680 a	690 c	690 c
Moisture content	11.00 b	9.08 a	8.87 a	11.16 d	8.14 c
Water absorption 2 h	69.10 a	61.33 a	50.42 a	80.67 c	39.90 b
Water absorption 6 h	77.57 a	58.72 a	71.94 a	89.69 c	49.13 b
Water absorption 24 h	84.31 a	70.78 a	84.00 a	96.39 c	63.01 b
Water absorption 48 h	87.32 ab	75.69 a	95.32 b	103.76 d	68.46 c
Water absorption 168 h	94.73 ab	82.66 a	101.25 b	115.81 d	69.95 c
Thickness swelling 2 h	8.88 a	7.34 a	8.35 a	11.62 d	4.77 c
Thickness swelling 6 h	12.75 b	9.03 a	10.52 ab	15.93 d	5.61 c
Thickness swelling 24 h	14.27 b	10.96 a	13.12 ab	17.81 d	7.76 c
Thickness swelling 48 h	16.20 a	14.49 a	15.72 a	21.71 d	9.22 c
Thickness swelling 168 h	18.56 a	16.34 a	19.44 a	26.53 d	9.69 c
ASE 24 h	45.37 a	43.24 a	58.83 a	-	-
ASE 48 h	51.11 a	69.68 a	65.07 a	-	-
ASE 168 h	51.13 a	71.06 b	70.18 b	-	-
Leaching	6.12 c	5.21 b	3.97 a	6.98 e	3.21 d
L*	62.03 b	58.10 a	59.56 ab	82.78 d	37.02 c
a*	3.10 b	1.70 a	1.76 a	1.57 d	2.80 c
b*	7.03 b	9.93 c	3.33 a	9.93 e	3.60 d
C*	8.44 a	10.15 b	7.25 a	10.12 d	7.11 c
H*	57.49 b	78.58 c	11.32 a	79.81 e	18.45 d
∆E	50.54 b	42.12 a	47.77 ab	-	-

Note: (a, b, c, d, e) a through e alphabet values followed by the same letter within row are not statistically different; (-) no data.

**Table 4:** Color characteristics of rattan particleboard.

Parameter	PF		MF		UF	
	K	FA	K	FA	K	FA
L*	86.87 (7.96)	37.2 (8.65)	79.07 (5.39)	37.13 (4.73)	82.4 (5.62)	36.73 (5.7)
a*	1.40 (0.74)	4.80 (1.21)	1.87 (0.92)	1.53 (0.52)	1.47 (0.64)	2.07 (1.22)
b*	11.00 (1.85)	3.07 (1.10)	8.67 (3.06)	11.2 (2.11)	10.13 (2.23)	−3.47 (1.55)
Chroma/C*	11.12 (1.8)	5.76 (1.39)	8.98 (2.84)	11.32 (2.07)	10.26 (2.24)	4.25 (1.42)
Hue/H*	82.43 (4.58)	32.56 (8.25)	75.35 (11.40)	81.83 (3.60)	81.67 (3.37)	−59.03 (18.08)
∆E	-	50.54 (9.49)	-	42.12 (6.36)	-	47.78 (8.02)
Hexadecimal color	#ecd4d7	#6b5249	#dbbeb4	#675255	#e5c7bd	#6a5148
Color description	Light grayish red	Very dark grayish orange	Light grayish orange	Very dark grayish red	Light grayish orange	Very dark grayish orange

**Table 5:** Variance analysis of the mechanical properties of rattan particleboard.

Parameter	Adhesive Type			Treatment		
	F	df	*p*-Value	F	df	*p*-Value
Modulus of elasticity	2.89	2	0.07 ns	4.19	1	0.06 ns
Modulus of rupture	3.14	2	0.06 ns	7.10	1	0.01 **
Internal bonding	3.06	2	0.07 ns	7.75	1	0.01 **
Hardness	6.44	2	0.01 **	4.89	1	0.04 **
Screw holding power	7.74	2	0.00 **	9.27	1	0.00 **

Note: (**) indicates highly significant differences at *p* ≤ 0.01; (ns); not significant; (-) no data.

**Table 6:** Duncan’s multiple range test of physical properties rattan particleboard.

Parameter	Adhesive Type			Treatment	
	PF	MF	UF	Control	FA
Modulus of elasticity	626 a	812 b	788 ab	671 c	813 d
Modulus of rupture	7.04 a	9.56 b	9.09 ab	9.73 d	7.40 c
Internal bonding	0.11 a	0.22 a	0.10 a	0.08 b	0.21 c
Hardness	6876 ab	7877 b	5399 a	6090 c	7345 d
Screw holding power	286 a	408 b	382 b	318 c	399 d

Note: (a, b, c, d) a through d alphabet values followed by the same letter within row are not statistically different; (-) no data.

## Data Availability

The original contributions presented in this study are included in the article. Further inquiries can be directed to the corresponding authors.
